# Chicago Med. Society—Paper on the Obstetric Forceps

**Published:** 1875-02

**Authors:** Philip Adolphus


### Reports of Societies.

### CHICAGO MEDICAL SOCIETY.

### THE OBSTETRIC FORCEPS.

(Read before the Society, at its meeting held December 8, 1874.)

By PHILIP ADOLPHUS, M.D.

This paper will treat of the obstetric forceps.

We will not attempt an exhaustive article, but speak of those points only on which authorities differ. We will examine the subject, pointing out errors having the endorsement of eminent men, and stating the latest practical reforms.

A critical analysis of different authors will discover many conflicting directions; author has copied author, until an assertion or theory, unsupported by fact, has, by constant repetition, assumed the force of law. Much has been presented in an abstruse form, calculated to intimidate the physician, and restrain him from rendering assistance at the proper period.

Demonstrations on the "Phantom" had evidently given birth to rules which cannot be adopted in deliveries, without injuring the woman. Thus, the single curved forceps was first employed; and authors taught that the blades should always be applied "to the os parietal, and extending on either side, in the direction of the occipito-mental diameter of the head" (Bedford); whether the head presented antero-posteriorly, obliquely or transversely. When Levret and Smellie added the sacral curve to the instrument, most authorities, amongst whom we may note Simpson, Dewees, Meigs, Hodge, Bedford and Elliot, (although the double curve of the instrument was adapted solely to the sacral curve of the pelvis), still maintained that the blades of the forceps should be applied to the lateral portion of the child's head, regardless of its position in the pelvis.

Thus they teach even to-day, that the head in a transverse position, in the superior strait, or cavity, or inferior strait, should be grasped by the forceps, with one blade applied in front of the sacrum, and the other in front of the symphysis pubis.

Moreover, eminent men, inexperienced in the use of the forceps, were afraid of its general introduction.

Thus, in Dr. Joseph Clarke's mastership of the Rotunda Hospital, Dublin, from 1787 to 1792, during which 10,287 deliveries took place, the forceps were used only 14 times; and in his private practice, extending over a period of forty years, during which he attended 2,878 labors, Dr. Clarke only once attempted to apply the forceps, and then did not succeed. (Collins' "Practical Midwifery," fol. 18.)

In the same hospital, under the mastership of Dr. Charles Johnson, from 1842 to 1845, in 6,702 deliveries, the forceps were only used in 18 cases.

I may give the statistics of the Royal Maternity Charity, as quoted by Dr. Ramsbotham, where, out of 48,996 deliveries, forceps, long and short, were used 73 times, or once in 671.2 cases. Consequently, the English became experts in embryotomy, and the lives of mothers and children were sacrificed, in just such cases, where the French, and especially the Germans, used the forceps. A glance at Churchhill's statistics will illustrate this.

In forceps cases 1 child in 5 dies, and 1 mother in 34; craniotomy necessarily destroys all the children, whilst the maternal mortality is estimated as 1 in 5.

Of late the double curved forceps are used where the perforator was formerly employed; nevertheless, the statistics of English and Continental midwifery, on this subject, show a marked difference in favor of the Germans.

The frequency of forceps cases has been estimated by Churchhill to be, amongst British practitioners, about 1 in 249; French, 1 in 140; and German, 1 in 106.

Churchhill lays down the rule, that forceps are to be applied in no case, till we are perfectly satisfied that the obstacle cannot be overcome by the natural powers with safety to the mother and child, and in this opinion he is upheld by most authorities.

Dr. C. C. P. Clark, in Trans. N. Y. Med. Society, 1870, comments on this as follows : "It is such a rule as this, causing perilous delay, that makes this instrument, in crude statistical tables, seem the means of death."

The time will arrive, and it is foreshadowed now in the practice of not a few practitioners, when the forceps will be used—in cases where delivery would have terminated by the natural powers—whenever the second stage of labor ceases to be actively progressive. By this procedure, much anxiety, pain and exhaustion is spared the mother. It is especially indicated in primapara, where, owing to delay, so many children are still-born.

Prof. Thos. Savage (in British Med. Journal, Aug. 14, 1869, page 183,) illustrates this point. The following table shows his practice :

| | | | | | | |
|---|---|---|---|---|---|---|
| In 1864, | out of | 73 | labors, forceps were used | 3 | times, or | 1 in $24\frac{1}{3}$ labors. |
| " 1865, | " | 154 | " " " " | 15 | " " | 1 in $10\frac{1}{3}$ " |
| " 1866, | " | 173 | " " " " | 18 | " " | 1 in $9\frac{2}{3}$ " |
| " 1867, | " | 203 | " " " " | 37 | " " | 1 in $5\frac{2}{3}$ " |
| " 1868, | " | 204 | " " " " | 31 | " " | 1 in $6\frac{2}{3}$ " |
| Total, | | 807 | labors, in which forceps were used | 104 | times. | |

Of these 807 cases, five children were still-born, and one maternal death occurred from puerperal fever. He states that in none of his cases has he found any ill effects afterwards, such as laceration of the soft parts, or extensive rupture of the perineum.

The best forceps for the general practitioner are those that can be used whenever a suitable indication for the delivery of the child presents itself, whatever the presentation of the head, and its position in the pelvis.

We therefore would reject the single curved forceps, because the shape does not accommodate itself to the direction and form of the pelvic axes ; for it can be used only when the head is low down in the excavation, or close to the perineum.

The double forceps should be chosen, for they can be applied to any portion of the pelvis, the leverage is greater, and any operation pertaining to this instrument can be performed with it. The possession of one forceps only is necessary. The multiplicity of instruments is a sign of weakness. The use of one instrument, perfectly adapted to the work it has to perform, is always a desideratum. Simpson, Hodge, as well as Barnes, advise the rejection of the short forceps, for the more efficient and powerful long instrument.

Therefore, whenever in this paper the forceps are mentioned, we do not allude to the short instrument, but to the long double curved forceps.

It is our duty to use the forceps, "whenever in a head presentation, with probable room for the head to traverse the pelvis, and with the os fully dilated, or partly dilated, and easily dilatable, the longer continuance of unaided labor involves danger either to the mother or the child."*

The forceps should also receive a tentative but patient trial in cases which require the mutilation of the fœtus; for the indications are often not to be distinguished, and can only be determined by trial.

Even symptoms *pointing towards* exhaustion, impaction and contusion, and *not merely the actual state* of exhaustion, collapse, contusion and inflammation, call for the application of the forceps.

Time as an element must not be considered: the state of the patient determines our action.

For a woman must be conducted through a labor, natural or instrumental, without a dangerous strain on her system. She must be relieved artificially, whenever, in spite of the administration of sedatives, the effects of irritation show themselves.

The administration of anæsthetics in forceps labors should be avoided, especially where long continued steady tractions are necessary to relieve the mother. It is a satisfaction in these cases, even to exceptionally skillful and experienced men, to be able to ascertain, by the expression of the patient's countenance, that no in-

* C. C. P. Clarke, op. cit.

jury is inflicted by the instrument. Besides, in just such cases, the non-administration of anæsthetics gives the child an additional chance of safety.

The forceps adapt themselves to the anatomical condition of the pelvis and soft parts, not to the position of the head. The blades of the forceps are fashioned to accord with the axes of the pelvis ; their sacral curves must follow and coincide with the utero-vaginal curve ; therefore, they must be introduced, placed into, and removed from within, the pelvis according to those laws. Any other course leads to the injury of the mother's parts.

In fact, authors who order the forceps to be placed along the child's head, place them, after locking, on the sides of the pelvis ; for they insist (I quote Bedford's words, see fol. 56, Edit. 1871) that "*the head being in a diagonal position, after locking the instrument, and before making any extractive force, the first thing to be done is, gently to turn the forceps, for the purpose of producing the movement of rotation, which will necessarily change the head from the diagonal to the direct position.*" These directions are placed in italics, and he adds: "Many a child has been sacrificed, and the mother cruelly lacerated, from the neglect of this fundamental principle in delivery by forceps." Ramsbotham, however, states that when the impediment of the brim is overcome, the head turns of its own accord with face towards the hollow of the sacrum, *without the use of any directing power on our part.*

The same author says (Med. Times and Gazette, 1862): "In employing the short forceps, I lay it down as a rule, that the blades should be passed over the ears, the head is more under command when embraced laterally, and there is less danger of injuring the soft parts during extraction." He, however, adds: "*But I confess that I have for many years been accustomed, however low the head may be, to introduce the blades within each ilium,*

*because they usually pass up more readily in that direction.*"

Dr. Barnes, commenting on this passage in his "Obstetrical Operations," says: I think I am not wrong in believing that many others do the same thing, some not knowing it, and even imagining that they are following the ancient rule. Therefore it is to the sides of the *pelvis*, and not to the sides of the *head*, that the blades of the forceps should be applied. If the child's head is placed diagonally, one blade, on being laid within each ilium, will fit the forehead, the other will apply itself to the occiput. The correctness of this assertion is proved by inspection of the head, after delivery, when the impress of the blades of the forceps over one of the oblique diameters of the fœtal head will be seen, and almost never along the parietal bones.

We herein follow the authority of the German accoucheurs, quoted by Cazeaux (fol. 69, ed. 1868), who recommend the blades to be placed on the sides of the pelvis in all cases.

This proposition once established, what amount of ambiguity, complexity and false teaching vanish? What becomes of the variations of application of this instrument, in the different positions of the head, at the inlet, cavity and outlet, which are taught us by authors? These may be practiced on the Phantom, but would crush the soft parts of the parturient patient.

Therefore place the patient on the back, as directed in the books, take the blades of the forceps, join them, and holding the instrument with the concavity of the pelvic curve forward, and the blades in the position they ought to occupy in the pelvis.

Consider, that you are about to embrace between them the globular head of the fœtus, which you know is within the pelvis; that you therefore must pass the separated blades within the pelvis, according to correct anatomical principles; placing one within the left and the other within the right ilium, the convex portion of the forceps fitting the concave portion of the sacrum. Consider also that, having placed them opposite each other, you have to lock them; and after locking, the two blades with the head of the child must become a unity. Thus, the handles being grasped, the child is to be extracted by following the anatomical curve of the sacrum, vagina and perineum.

Then proceed to separate the warmed and oiled blades, and take the left or male blade near the centre, into your left hand, and introduce it into the vulva, slip it along the two fingers of the right hand, put between the cervix uteri and the child's head: it will guide itself and be placed alongside the left lateral portion of the pelvis, embracing the head. Leaving it in the hands of an assistant, take the right or female blade into your right hand, introduce it into the vulva, along the palps of the fingers of the left hand, which are placed between the os uteri and the child's head, and it will guide itself along and within the right lateral portion of the pelvis, and these embrace that part of the head which it will find there.

*Do not look for any particular portion of the child's head; disregard its position, but be sure to regard the anatomy of the hard and soft parts of the pelvis.*

Your blades, thus anatomically conducted, will enter easily, instinctively and unerringly, and will place themselves on each side of the globular head, which they will embrace.

Do not watch the handles of your forceps, as the books teach you, do not burden your memories with a thousand details, which you will forget at the bedside. Let your brain have a thorough conception of the anatomy of the pelvis, its containing head, the adapting curves of the forceps, and the act you are to perform: the hand will then execute the bidding of the will, when the blades of the forceps disappear from sight.

Your forceps will lock easily, provided you have met with no resistance during their introduction. If you meet with it, you must overcome it by a gentle backward, upward and inward movement, which will result in placing the blades fairly upon the head, at the same time carrying them back into the axis of that part of the utero-vaginal canal in which the head is situated.

If you cannot lock the forceps, after fair and skillful trial, remove the blades, examine the pelvis, look for deformities, and determine between turning and craniotomy.

Then extract, steadily, an assistant pressing the retreating uterus downwards. Rest, during the absence of a pain; extract according to the anatomical direction of the sacrum, vagina and perineum, until the child is born.

Authors tell us, whilst the head is distending the perineum, during the use of the forceps, to sustain it, or have it sustained. The practitioner cannot extract in difficult cases, and support the perineum at the same time. Moreover, assistants, skilled or otherwise, obscure the parts, and prevent the practitioner from seeing and feeling the amount of distention. There is less chance of its rupture in forceps operations, for the perineum is distended gradually, intelligently and anatomically, by the will of the physician. Should, however, a predisposition to laceration exist, it cannot be avoided.

How much traction and pressure you can apply in the extraction of the head cannot be taught. In ordinary cases, as little as possible; in difficult cases, as much as experience and intuition will teach. It may be necessary, in extreme cases, (namely in occipito-posterior positions, which are further complicated by disproportion between the pelvis and the head) to brace the foot against the sides of the bed, to pull with your arms (and not with your back) until overcome by sheer exhaustion. Some authorities call this bad practice, but it is not so, for it has saved many a child from the crotchet.

To use the words of Elliot, "You may find that just such tractions are the only ones which can terminate a labor without recourse to embryotomy; and that just such tractions, repeated again if necessary, may alone justify the subsequent resort to embryotomy."

Is the use of the forceps dangerous?

In cases where no deformity of the pelvis exists, when the head has rotated under the arch of the pubis, or when it is within the pelvis, the use of the forceps is without danger to mother and child. Gentle tractions, with an occasional oscillation, will suffice to deliver the head. These represent the majority of cases.

There is danger of bruising the soft parts of the mother, in the use of the forceps.

1st. We should be careful when applying the blades, to introduce them within the os uteri, and not between the vagina and the neck of the womb. We should feel for the edge of the os uteri, place the palps of our fingers on it, and, guarded by these, slip the blade of the forceps within the os uteri; else we perforate the cul-de-sac of vagina, and penetrate into the peritoneum.

2nd. If we follow the directions of reputed authors, and apply the long forceps to the sides of the head, when according to Byford, "it is situated diagonally with reference to pelvic curves, we incur much danger of touching the sides of the pelvis with the end of the blades, and bringing them in contact with the ramus of the ischium, at the outlet, and thus dangerously contusing the soft parts at these points." He adds, "I think, indeed, that there is very much more damage done to the soft parts of the mother, by making the diagonal application of the curved forceps, than there would be, if the rule was to use them with reference to the pelvic curves entirely." (Byford, fol. 317, ed. 1873.)

3rd. The direction of Tarnier and others, to correct a transverse or posterior occipital position, by rotating the forceps, after having applied it to the sides of the head, upon its own axis, in order to turn the head in the cavity of the pelvis from behind forward, bringing it first to the side of the pelvis and finally behind the pubis, *should be condemned.* If followed, extensive lacerations, contusions, and their consequences must result. These cases should be left to nature, for according to Miller and Leishman, anterior rotation will in most instances be effected. Should this not occur, apply the forceps on the sides of the pelvis, and deliver with the occiput posteriorly.

4th. Should great obstacles to the delivery of the head exist, traction, as we have already stated, must be increased ; the greater the traction, the greater the pressure. Increased pressure means danger to the child's life. Accordingly we have, as described by Schroeder, superficial dermatitis, suggillations and even circumscribed gangrene externally, and lacerations of the venous sinuses, and of other bloodvessels of the cranium within, which often produce the death of the child. Yet the use of force is justifiable, and is the only choice left the operator ; craniotomy would otherwise have to be performed. Hodge's remarks on this point are so just that I will reproduce them here ; and his views on this point are endorsed by Dewees, Barnes, Elliot, and Schroeder. He says : "our practice must be regulated not so much by the idea how much compression can be borne with impunity, but how much is absolutely demanded to accomplish delivery ;" and Elliot states that great traction and compression offer the child a chance that the perforator can never offer, and therein lies its advantage.

In concluding, a few words may be said of the use of the forceps, when the head is above the superior strait.

The forceps ought not to be used when the head is floating and movable above the superior strait. We should wait until it becomes wedged into the pelvis. "In the contracted pelvis, the use of the forceps is contra-indicated until the head has passed through the contracted brim. (Schroeder, fol. 178, ed. 1874.)

Should the life of the mother and the child be endangered by convulsions, hemorrhage, prolapse of the cord, or excessive volume of the head, we must turn and deliver. Almost all authorities are agreed on this point, yet every one gives the rules of procedure of a plan which they condemn.

---

Dr. Paoli: The paper is well prepared, but better adapted to be read before a class of students than before a society of physicians; reports of cases from every day experience would be of more profit to the Society.

Dr. Earle: Favors the use of forceps as often as necessary, but objects to their employment as a means of saving time. Hemorrhage is more likely to occur where forceps are used and delivery hastened, and it is desirable to make pressure on the abdomen following the contracting uterus as the child is being withdrawn.

Dr. Bridge: Had used pressure over uterus to facilitate labor without the success he was led to expect from the advocates of this method. He agreed with the author, and believed that the use of forceps was the subject of the day and hour in obstetrics.

Dr. Quine: Concurs with the author's views except in that he believes it practicable and advisable to apply the forceps in some cases before the head is engaged in the superior strait; he has used them twice in this way, and without encountering great difficulty. The instruments may be used oftener than there is need, but it is better to err in this direction than to be too hesitating in their employment. Forceps may be used to obviate laceration of the perineum. It is not so difficult as it seems to be imagined to adjust the forceps before the head is engaged in the superior strait, and in such cases where there is an urgent indication for speedy delivery, he considers it his duty to try the forceps before resorting to podalic version.

Dr. Adolphus: The uterus, if foiled, works itself into a fury and tends to produce laceration of the perineum, but we may by the forceps regulate the degree of tension of the soft parts. Forceps are not used as often as they should be in this country. Where the contractions, from their energy and frequency, render the occurrence of laceration probable, he is in the habit of prescribing morphine, sometimes with and sometimes without ant. et pot. tart. When a young man fails in the application of the forceps it is the fault of his teacher, he prefers to make version rather than attempt to use forceps before the head is engaged in the superior strait.

Dr. Hutchinson: Used forceps without hesitation when it was necessary to do so; he applied them in a common sense way and *held on*, and he considers the directions for their application to particular parts of the child's head to be unworthy of serious consideration.

Dr. Pearson: Considers the use of forceps above the superior strait preferable to version. Toward the close of the meeting this gentleman tendered his resignation as a member of the Society, which was accepted.

